# High-order harmonics generation in the laser-induced lead-free perovskites-containing plasmas

**DOI:** 10.1038/s41598-022-13010-w

**Published:** 2022-06-01

**Authors:** Vyacheslav V. Kim, Rashid A. Ganeev, Srinivasa Rao Konda, Ganjaboy S. Boltaev, Ibrohim B. Sapaev, Weili Yu, Wei Li

**Affiliations:** 1 GPL Photonics Lab, State Key Laboratory of Applied Optics, Fine Mechanics and Physics, Changchun Institute of Optics, Chinese Academy of Sciences, Changchun, 130033 China; 2 Laboratory of Nonlinear Optics, Institute of Astronomy, University of Latvia, Jelgavas iela 3, Riga, 1004 LV Latvia; 3 Department of Physics, Voronezh State University, Voronezh, 394006 Russia; 4 Tashkent Institute of Irrigation and Agricultural Mechanization Engineers, National Research University, Kori Niyozov street 39, Tashkent, Uzbekistan 100000; 5 Akfa University, Milliy Bog Street 264, Tashkent, 111221 Uzbekistan

**Keywords:** Optics and photonics, Physics

## Abstract

High-order harmonics generation in the laser-induced plasmas produced on the surfaces of lead-free perovskites is studied. We analyze the harmonics generation in (CH_3_NH_3_)_2_CuCl_4_ and (CH_3_NH_3_)_2_CuBr_4_ plasmas during their ablation by the femtosecond, picosecond, and nanosecond pulses. The modifications of the high-order harmonics spectra are studied using the -color pump scheme (800 nm and 400 nm, 40 fs pulses). The influence of the variations of laser chirp and pulse duration on the dynamics of high-order harmonics generation is examined. The spectral shift, chirp-related harmonic cutoff scaling, and the role of the pulse duration of converting and heating laser radiation are examined at different conditions of plasma formation and harmonic generation. The dependencies of the pulse duration and the fluence of heating pulses on the harmonic’s blue shift are found. The effect of harmonics broadening and splitting on the two red- and blue-shifted components is demonstrated.

## Introduction

High-order harmonics generation (HHG) in the laser-induced plasmas (LIP) is a simple way to analyze a diversity of materials by studying this nonlinear optical (NLO) process using a broad range of samples (from simple metals to complex molecular compounds)^1–6^. Additionally, the combination of different methods like application of two and multi-color pumps^7,8^, probing extended and multi-jet plasmas in the frame of the quasi-phase-matching approach^9^, time-resolved LIP probing^10^, searching for the advanced nanostructured plasmas^11–13^, resonance enhancement of generation harmonics^14–21^, search for the advanced molecular plasmas for efficient HHG^22,23^, analysis of the spatial coherence of harmonics from plasmas^24^, generation of attosecond pulses during HHG in LIP^2,25^, HHG spectroscopy of materials^26^, application of vortex beams for HHG in plasmas^27^, etc. enriched the range of the phenomena involved in this field of study. Thus the HHG in LIP can serve as the advanced tool for analyzing the properties of various materials.

Recently, a new class of materials united under the common term ‘perovskites’ attracted intensive research attention due to their exciting optical and optoelectronic properties^28–31^. These materials have potential applications in lasers, light-emitting diodes, photodiodes, and photodetectors. Perovskite materials have also proven to be excellent NLO materials in a broad spectral range thus making them the promising candidates for photonics and optoelectronics applications. NLO properties such as saturable absorption, reverse saturable absorption, two-photon-absorption process, and third-order NLO properties have been demonstrated^32–44^ for the metal-containing perovskites. However, the high-order NLO properties of perovskite materials are still demanding further research. Recently, high-order NLO properties via HHG in LIP of Ni-doped CsPbBr_3_ colloidal nanocrystals were investigated and the enhancement of harmonics was demonstrated^45,46^. Meanwhile, an alternative class of lead-free perovskites like MA_2_CuCl_4_, MA_2_CuBr_4_, etc., where Pb^2+^ ion became substituted by Cu^2+^, is of primary importance due to lower toxicity. Here MA is referred to as the methylammonium (CH_3_NH_3_).

In this paper, the high-order NLO properties of the two lead-free perovskite materials MA_2_CuCl_4_ and MA_2_CuBr_4_ are investigated. The presence of Cl is essential to improve the material stability against copper reduction and enhance the perovskite crystallization for the family of perovskite materials with the common chemical formula MA_2_CuCl_x_Br_4-x_ where x changes from 0 to 4. While MA_2_CuCl_4_ is monoclinic, the materials with mixed halides MA_2_CuBr_4_ crystallize with an orthorhombic crystal system. The difference between two perovskite materials was shown through the HHG under the influence of the strong electromagnetic field.

**Figure 1:**
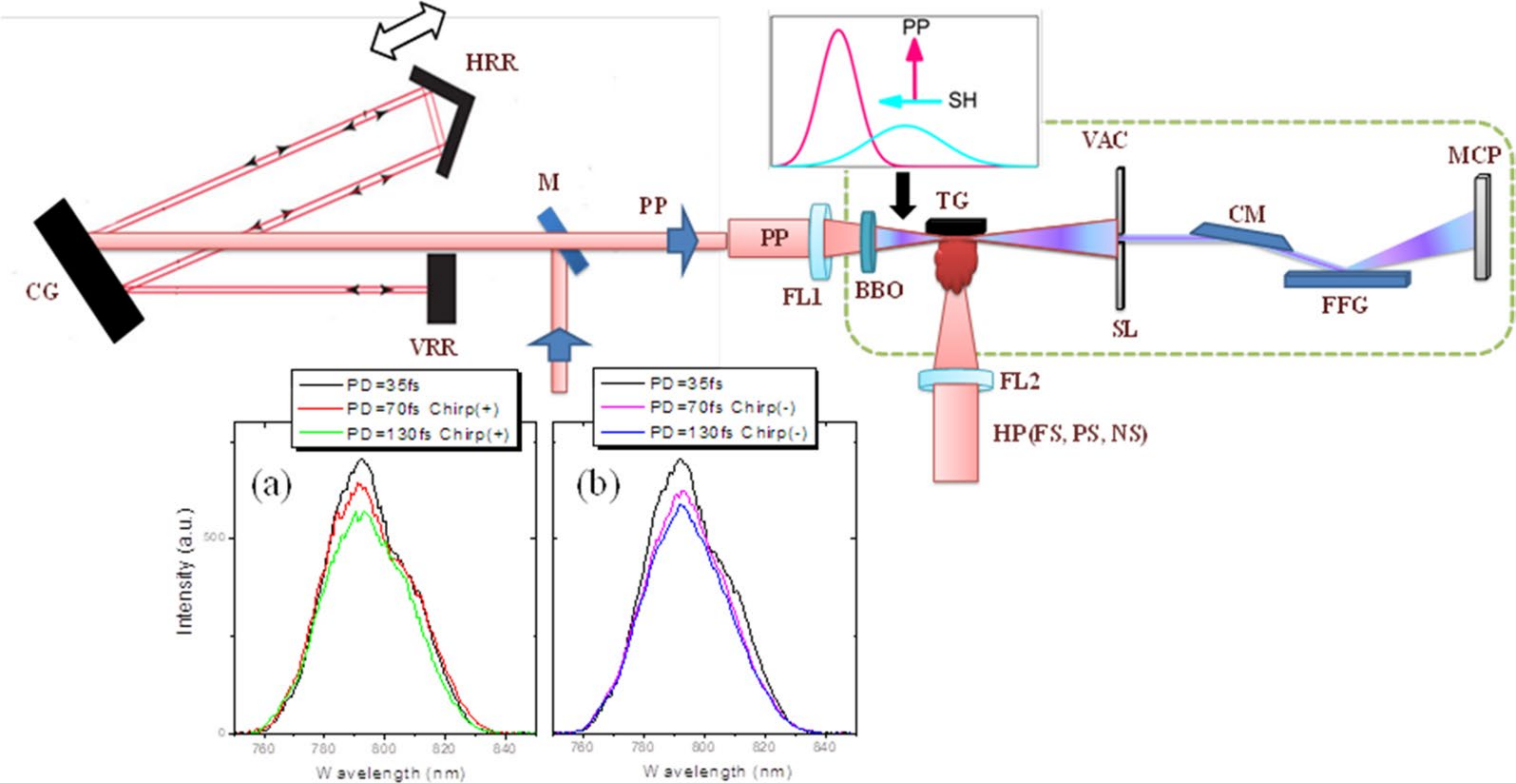
The schematic view of HHG setup. The uncompressed laser pulse was directed by a mirror (M) to the compressor comprising of the compressing grating (CG), vertical retroreflector (VRR), and horizontal retroreflector (HRR). The compressed probing pulse (PP) was focused by a focusing lens (FL1) inside the vacuum chamber (VAC) comprising the target and XUV spectrometer. The focused pulses propagated through the 0.2 mm thick BBO crystal to generate second harmonic (SH) for the two-color pump of plasma. Inset shows the relative positions of the SH (blue curve) and PP (red curve) pulses and their polarizations. The heating pulses (HP) were focused by a focusing lens (FL2) to produce the laser plasma on the surface of the target (TG). The generated harmonics and fundamental radiation propagated through the slit (SL) and entered the XUV spectrometer comprising of the cylindrical mirror (CM), flat-field grating (FFG), and micro-channel plate (MCP). Bottom panels: The spectra of (**a**) chirp-free 35 fs pulses and positively chirped 70 and 130 fs pulses and (**b**) chirp-free 35 fs pulses and negatively chirped 70 and 130 fs pulses. PD, pulse duration.

Here, the laser plasmas induced by femtosecond, picosecond, and nanosecond pulses were probed with positively/negatively chirped and chirp-free femtosecond two-color (fundamental and second harmonic (SH)) pulses (Fig. 1). We discuss the peculiarities of the HHG spectra at different conditions of experiments with those two perovskites.

## Results

**Figure 2:**
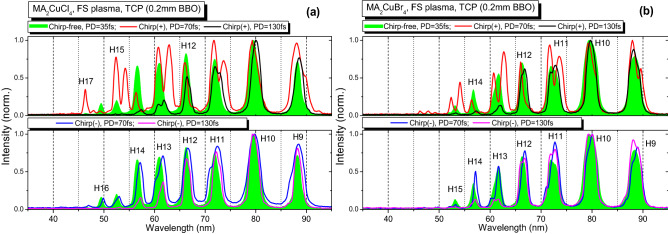
Harmonic spectra in the case of TCP scheme generated in two LIPs produced by FS HP. (**a**) HHG in MA_2_CuCl_4_ LIP. (**b**) HHG in MA_2_CuBr_4_ LIP. The harmonic spectra depending on the pulse duration and the sign of chirp are shown. Green filled profiles of harmonics correspond to the chirp-free 35 fs PP (Chirp-free, PD = 35 fs), red curves correspond to the positively chirped 70 fs PP (Chirp( +), PD = 70 fs), blue curves correspond to the negatively chirped 70 fs PP (Chirp(-), PD = 70 fs), black curves correspond to the positively chirped 130 fs PP (Chirp( +), PD = 130 fs), and purple curves correspond to the negatively chirped 130 fs PP (Chirp(-), PD = 130 fs).

**Figure 3:**
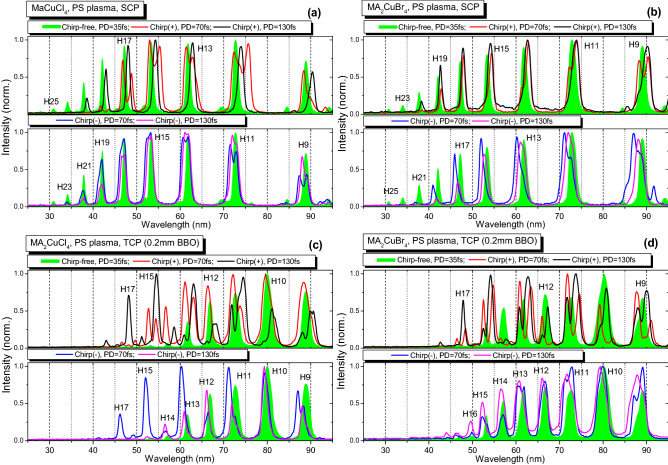
HHG spectra from two plasmas produced using the PS HP. Panels (**a**) and (**b**) correspond to SCP HHG in MA_2_CuCl_4_ LIP and MA_2_CuBr_4_ LIP respectively. TCP data are presented on the panels (**c**) HHG in MA_2_CuCl_4_ LIP and (**d**) HHG in MA_2_CuBr_4_ LIP. The harmonic spectra depending on the pulse duration and the sign of chirp are shown. Green filled profiles of harmonics correspond to the chirp-free 35 fs PP (Chirp-free, PD = 35 fs), red curves correspond to the positively chirped 70 fs PP (Chirp( +), PD = 70 fs), blue curves correspond to the negatively chirped 70 fs PP (Chirp(-), PD = 70 fs), black curves correspond to the positively chirped 130 fs PP (Chirp( +), PD = 130 fs), and purple curves correspond to the negatively chirped 130 fs PP (Chirp(-), PD = 130 fs).

**Figure 4:**
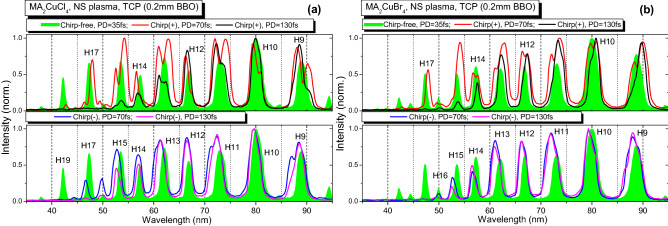
Harmonic spectra generated using TCP scheme in the LIPs of two ablated perovskites using NS HP. (**a**) HHG in MA_2_CuCl_4_ LIP. (**b**) HHG in MA_2_CuBr_4_ LIP. The TCP-induced spectra depending on the pulse duration and the sign of chirp are shown. Green filled profiles of harmonics correspond to the chirp-free 35 fs PP (Chirp-free, PD = 35 fs), red curves correspond to the positively chirped 70 fs PP (Chirp( +), PD = 70 fs), blue curves correspond to the negatively chirped 70 fs PP (Chirp(-), PD = 70 fs), black curves correspond to the positively chirped 130 fs PP (Chirp( +), PD = 130 fs), and purple curves correspond to the negatively chirped 130 fs PP (Chirp(-), PD = 130 fs).

Below we present HHG spectra from the studied LIPs while using different pulse durations of probing pulses (PP) and heating pulses (HP), signs of the chirped PP, and material type. Figures 2, 3, and 4 comprise the extreme ultraviolet (XUV) spectra generated during two-color pump (TCP) of the LIP produced by the heating pulses of different duration (from 35 fs to 5 ns). In the case of femtosecond heating pulses (FS HP, Fig. 2) and picosecond heating pulses (PS HP, Fig. 3), the delay between HP and PP was fixed and equal to 70 ns. The fluence of HP was chosen to achieve the highest cut-off and harmonic yield.

During these experiments, we found that, in the case of nanosecond heating pulses (NS HP), the optimal fluence was the highest compared with the case of the shorter durations of HP. The corresponding fluencies were ~ 0.6 J cm^-2^ for the FS HP and ~ 2 J cm^-2^ for the PS HP. The pulse duration was fixed for PS HP and equal to 200 ps, while FS HP varied together with the PP duration between 35 and 130 fs. As it was pointed out in the previous section, in the case of 5 ns HP (Fig. 4), we determined the optimal delay between HP and PP. In that case, the pair of optimal parameters, fluence, and delay for NS HP, was correspondingly equal to ~ 9 J cm^-2^ and 400 ns.

For all HP, the same configuration of PP was used. LIPs were probed using the 35, 70, and 130 fs pulses. The last two options were maintained by adjusting the distance of the beam path in the compressor stage of the laser. The same pulse duration was maintained at two positions of the compressor corresponding to the positively and negatively chirped 70 fs or 130 fs pulses. The fluence of the fundamental 800 nm PP was kept the same (~ 11 J cm^-2^), while the intensity of those pulses (*I*_*ω*_) was varied depending on the pulse duration. For three pulse durations, we measured it as *I*_*ω*_ (35 fs) = 3 × 10^14^ W cm^-2^, *I*_*ω*_ (70 fs) = 1.5 × 10^14^ W cm^-2^, and *I*_*ω*_ (130 fs) = 8.4 × 10^13^ W cm^-2^.

The broadening of second harmonic (SH) pulses compared with the fundamental radiation and the delay between 400 and 800 nm pulses can be determined using the relations (1) and (2)^47^:

1 $$ \tau_{2\omega } = \left[ {\left( {\Delta_{delay} } \right)^{2} + 0.5\left( {\tau_{\omega } } \right)^{2} } \right]^{1/2} $$

2 $$ \Delta_{delay} = d\left[ {\frac{1}{{v_{\omega } }} - \frac{1}{{v_{2\omega } }}} \right] $$

Here *t*_2*ω*_ is the pulse duration of SH after propagation of the fundamental pulse through the barium borate (BBO) crystal of the thickness of *d*, *t*_*ω*_ is the pulse duration of the fundamental radiation, and *v*_*ω*_ and *v*_*2ω*_ are the group velocities of the ordinary fundamental and extraordinary SH pulses, respectively. The pulse durations of SH and relative positions of the fundamental (red curves) and SH (blue curves) pulses in the probing TCP according to the simplified formulas (1) and (2) are shown in Table 1 in the case of 0.2-mm thick BBO. As one can see, with increasing fundamental pulse duration, the second harmonic pulse duration also increases. The region where two pulses overlap is growing so that, at *τ*_*ω*_ = 130 fs, SH becomes completely overlapped by the fundamental beam.

**Table 1:** The pulse durations of the fundamental pulse (800 nm), SH (400 nm), and relative positions of the fundamental (red curves) and SH (blue curves) pulses in the probing two-color pulse in the case of 0.2-mm thick BBO. The magnitude of the SH component is artificially increased for the visual comparison with regard to the fundamental pulse.

*τ*_*ω*_	*τ*_*2ω*_	Relative positions of pulses
35 fs	47 fs	
70 fs	64 fs	
130 fs	100 fs	

The presence of the relatively weak SH field (2*ω*) leads to the appearance of even harmonics, alongside the odd harmonics (2*n* + 1)*ω* of the fundamental radiation generating at the frequency *ω*. If one considers the independent contribution from the *ω* and 2*ω* fields, the generation of the odd orders from each field, (2*n* + 1)*ω* and (2*n* + 1)(2*ω*), could be expected. Such distinction between the two pumps has been demonstrated in^7^ in the case of TCP using the 0.7 mm thick BBO crystal. In that case, the thick crystal introduced the large Δ_delay_ between two pulses, strongly restricting their overlap in the generating media. If we use thin BBO crystals, Δ_delay_ will be small enough to overlap two pulses in LIP. Correspondingly, the harmonics of all orders of fundamental radiation appear in the HHG spectrum despite a significantly smaller SH conversion efficiency compared to the thick crystals.

Additionally, another consideration should be taken into account. As mentioned, the intensity of SH pulses even for the shortest pulse duration used in our experiments using 0.2 mm thick BBO was *I*_2*ω*_ ~ 1.2 × 10^13^ W cm^-2^. At these parameters, *γ*_*k*_ > 1, where *γ*_*k*_ is a Keldysh parameter^48^ and the HHG in the case of the 2*ω* PP cannot be considered as a tunnel process but rather a multi-photon one. Correspondingly, the three-step relation^49^:

3 $$ N_{c} = \left( {I_{p} + 3.17U_{p} } \right)/\omega_{0} $$

cannot be applied to determine the order of cutoff harmonic (*N*_c_). Here *I*_p_ is the ionization potential of the generating particle, *U*_p_ is the ponderomotive potential and *ω*_0_ is the PP frequency. The plateau-like picture of the HHG spectrum with *γ*_*k*_ > 1 is not realized. Instead, one can expect a fast decrease of (2*n* + 1)(2*ω*) harmonics yield according to the relation *I*_*H*_ ~ (*I*_2*ω*_)^2*n*+1^ rule, where *I*_*H*_ is the harmonic intensity. However, as it was demonstrated in^7^, the mixing of extremely weak SH field with fundamental beam (with the ratio of energies of 1:50) in the generating medium is sufficient to drastically change a picture of harmonics distribution, so one can identify a plateau-like picture for the even orders of harmonics in the spectrum. Thus, some harmonic orders like 12H, 16H, and 20H, which we observed in our experiments, cannot be attributed to any odd series set from the *ω* and 2*ω* pumps and point out the symmetry modification during TCP HHG. Below, we demonstrate this peculiarity of HHG using TCP at the conditions of chirp-free and chirped pulses propagating through the two perovskite-containing plasmas.

Chirp variation leads to the change of the PP pulse duration and better overlap with the SH component, as shown in Table 1. The increasing pulse duration while keeping the same pulse energy affects the intensity of PP. According to Eq. (3), this pulse broadening should lead to the change of the *N*_*c*_. The maximum cutoff order achieved in the case of the fundamental chirp-free 800 nm single color pump (SCP) was the same (25H, 38.7 eV) for both MA_2_CuCl_4_ and MA_2_CuBr_4_ samples (see panels (a) and (b) in Fig. 3) during the experiments using the FS, PS, and NS heating pulses. In the case of the chirp-free pulses (green filled profiles in the following Figs. 2, 3 and 4), the TCP (800 nm + 400 nm) changed the harmonics distribution and *N*_*c*_. In the case of both materials for all types of HP, the cutoff position moves towards the smaller harmonics orders. This behavior could be attributed to the increasing contribution of the short quantum trajectories of the accelerated electrons^50^.

In the case of TCP, the HHG in NS-produced LIP demonstrated an increase of cutoff position up to 21H (32.5 eV, Fig. 4, green filled profiles) as a result of the additional optimization of the delay between NS HP and FS PP towards the larger values (400 ns). In contrast, *N*_*c*_ position for SCP does not depend on the duration of HP. Similar to^7^, the presence of the SH field led to the appearance of the plateau-like distribution of even harmonics between the 10H to 18H orders. The comparison of chirp-free TCP HHG (Figs. 2, 3 and 4, green profiles) shows a similarity of harmonic spectra for shorter FS and PS heating pulses for the MA_2_CuCl_4_, MA_2_CuBr_4_ LIPs.

Meanwhile, in the case of the positively or negatively chirped PP, the notable variations in the harmonic spectral shifts, broadening, intensity redistribution and variations of the cutoff position were observed. The harmonic spectra in the case of the positively chirped 70 fs and 130 fs pulses in the case of SCP are shown on the top panels of Figs. 2, 3 and 4. They marked as the red (Chirp( +), PD = 70 fs) and black (Chirp( +), PD = 130 fs) solid curves. The HHG spectra in the case of the negatively chirped pulses (70 fs and 130 fs) are plotted on the bottom panels of Figs. 2, 3 and 4 as the blue (Chirp(-), PD = 70 fs) and purple (Chirp(-), PD = 130 fs) solid curves correspondingly.

Figure 2 shows the harmonic spectra generated in LIP using FS heating pulses and the delay between HP and PP equal to 70 ns. The ablation threshold of studied samples in the case of the femtosecond HP is lower due to higher instant intensity. And despite the lowest used fluence (~ 0.6 J cm^-2^) of HP, we observed the harmonics at approximately same intensity in comparison with the PS- and NS-induced plasmas ignited using the notably higher fluencies. This observation corroborates with^51^ where it was demonstrated that the FS LIP is significantly denser at smaller distances from the target surface compared to the NS LIP.

Applying the positively chirped 70 fs and 130 fs PPs led to the shift of harmonic spectra toward the longer-wavelength spectral range (red and black curves in Fig. 2a and b). In the case of the positively chirped 70 fs PP (red curves), the 11H, 13H, and 15H harmonics lines are broadened with the appearance of two, redder and bluer, components. The blue components coincide with the harmonics produced by chirp-free PP in the case of the MA_2_CuCl_4_ LIP (Fig. 2a, red curve) and even shifted towards the bluer side for MA_2_CuBr_4_ LIP (Fig. 2b, red curve). Meanwhile, the even-order harmonics (10H, 12H, and 14H) are shifted to the bluer part. It is clearly seen that they are generated by the blue component of the 800 nm PP. We also observed some increase of the cut-off position up to the 17H order.

In previous studies, the corresponding spectral shift was explained by the role of the leading part of pulse^52^. The harmonics are generally generated by the leading part of the laser pulses. Correspondingly, the abundance of either blue or red component of chirped pulse will lead to the shift of the harmonic wavelength towards the blue or red side with regard to the harmonics generating by chirp-free pulses. The splitting of harmonics lines could be the evidence of more complex behavior of the accelerating and recombining electron in the combination of the TCP and chirped pulses. This behavior can be explained by deviation from the above assumption related to the role of the leading part of the pulse. The broadening of harmonics differs from case to case. It is also a complex issue, since the value of this effect depends on various parameters of experiments.

Further increase of the chirp by using the 130 fs positively chirped pulses (Fig. 2, black curves) led to a smaller redshift of harmonics. We observed a reduction of the blue component compared with the case of the positively chirped 70 fs pulses. The even-order harmonics slightly shifted towards the redder side. The cut-off position was decreased, which could be explained by a fourfold decrease in the driving field intensity.

The application of negatively chirped 70 fs and 130 fs PPs (Fig. 2, blue and purple curves) led to entirely different harmonic spectra, contrary to the positively chirped pulses. We did not observe any expected blue shift while noting some redder shift of all harmonics. The cut-off position scaling was also different. The HHG spectra from two plasmas in the case of chirp-free 35 fs pulses and negatively chirped 70 fs pulses were almost equal (Fig. 2a and b, the green filed curves and blue curves). The cut-off slightly decreased only in the case of the longer (130 fs) negatively chirped pulses (Figs. 2a and b, purple curves).

Figure 3 shows the harmonics spectra generated in the LIPs ignited by PS HP (*t* = 200 ps) at a fluence of ~ 2 J cm^-2^. SCP and TCP HHG results are presented on the panels (a)–(d). The delay between HP and PP was the same (70 ns) as in the case of the above-described experiments using the FS HP. The optimal fluence of PS HP at which we achieved the maximal harmonic yield was found to be ~ 2 J cm^-2^. The effect of harmonics spectral modulation in the case of the positively chirped 70 fs and 130 fs PP (Fig. 3, red and black curves) similar to the case of FS HP (Fig. 2) was observed. The odd harmonics (9H – 15H) in the case of the positively chirped 70 fs PP (Fig. 3, red curve) were broadened and separated on the two (redder and bluer) spectral components. Note that the bluer components were stronger than the redder ones. Meanwhile, the even harmonics (10H – 14H) neither had the blue spectral shift nor showed the dominance of a bluer component (see for example, the spectrum of 10H from MA_2_CuCl_4_ LIP in the case of positively chirped 70 fs PP, Fig. 3c, upper panel, red curve).

At higher positive chirp and pulse duration (130 fs, Fig. 3, black curve), the red-shifted spectral components of the harmonics become dominant. Similar to the case of FS LIP, the positively-chirped PP increased the cut-off position (from 15 to 19H). However, in contrast to FS LIP, the longer (130 fs) pumping pulses produced higher cut-off harmonics despite the four-fold decrease of the driving intensity.

The behavior of the spectral shift of negatively chirped 70 fs and 130 fs PP (Fig. 3, blue and purple curves) was different compared with the case of FS-induced LIP (Fig. 2, blue and purple curves). In that case, we observed the blue shift of harmonics towards the shorter-wavelength spectral range without vital redder components. The variation of the laser chirp, in that case, allowed considerable tuning of the harmonics towards the shorter wavelengths (notably, by ~ 2.5 nm for the 13H and 15H; see Fig. 3c, bottom panel, blue curve).

Figure 4 presents the harmonic spectra from the two plasmas induced by the 5 ns pulses and highest fluence (~ 9 J cm^-2^) among three studied cases using different HP. While the delays between HP and PP for FS and PS heating pulses were equal to 70 ns and harmonic yield optimization was performed with fluence change, the application of the NS HP required an increase of delay up to 400 ns for the optimal conditions. This difference in optimal delays was caused by the slower formation and spreading of the plasma produced by 5 ns pulses. Similar to the two previous cases (FS and PS HP), positively and negatively chirped PP caused the red and blue spectral shifts of the harmonics. We also observed the broadening of odd harmonics in the case of the positively and negatively chirped PP (Fig. 4, red, blue and magenta curves) and splitting into the two spectral components. The specific feature of the HHG in NS LIP was the observation of the higher cut-off order for the chirp-free PP compared with the chirped PP.

**Figure 5:**
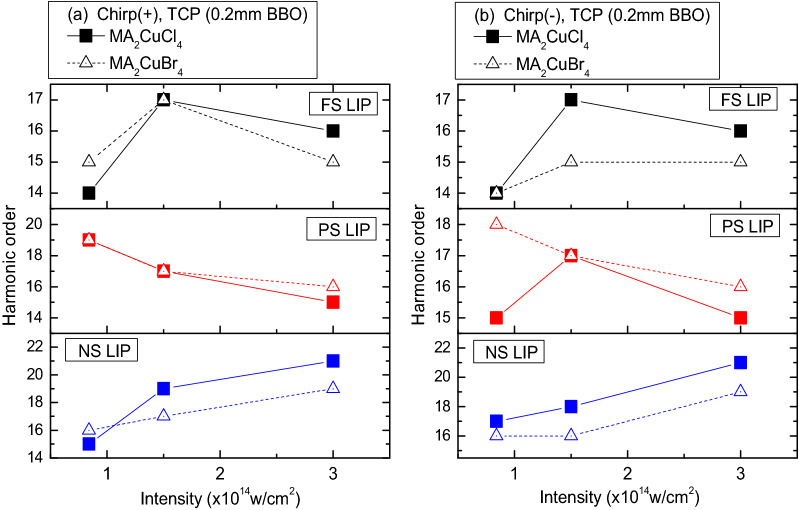
The cut-off *N*_*c*_ position dependence on the type of heating pulse and the chirp sign in case of the TCP scheme. X axis corresponds to 800 nm laser pulse intensity.

The variations of the cut-off position are summarized in Fig. 5. In the case TCP with chirped pulses, for FS and PS LIPs we observe a strong deviation from semiclassical rule defined by Eq. (3). With increasing the pump intensity *N*_*c*_ reaches maximum for 70 fs chirped two-color pulses (Fig. 5, FS LIP panels with black curves) and even decreases in the case of the PS LIP (Fig. 5, PS LIP panels with red curves). Only in case of the NS LIP we observed the “natural” behavior of cut-off position, close to the linear one, i.e. the increase of the cut-off order with pump intensity (Fig. 5, blue curves). This might be attributed to the higher delay time for NS HP (400 ns against 70 ns for FS and PS HPs). In the case of NS pulses, the laser induced plume has more time to expand, so the density of LIP becomes optimal.

Previously, the harmonics generation in LIPs from different metals^16,53,54^ and Ni-doped lead-containing perovskites^46^ were studied using the chirped SCP and TCP. The observed variation of the wavelength shift of harmonics with laser chirp was explained by the involvement in HHG of the spectral components presented in the leading front of the chirped laser pulse. The leading parts of negatively and positively chirped PPs produced the harmonics from the red and blue spectral components of the driving pulses, respectively, which led to the red and blue shifts of harmonics. These modifications of harmonic spectra can be used for application in the nonlinear spectroscopy of materials using tunable coherent XUV pulses.

**Figure 6:**
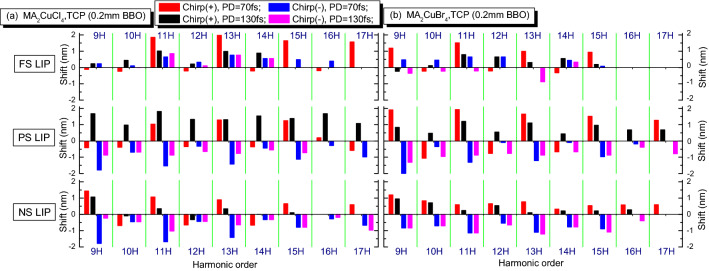
Spectral shifts (in nm) for the harmonics varying from 9 to 17H orders in the case of FS- to NS-induced LIPs for MA_2_CuCl_4_ (**a**) and MA_2_CuBr_4_ (**b**) perovskites in the case of TCP scheme. PD: pulse duration, Chirp( +): positively chirped pulses, Chirp(-): negatively chirped pulses.

The summarized spectral shifts of harmonics are presented in Fig. 6. We note that, in the case of two-component harmonics showing their splitting, the largest shift of the wavelengths of blue and red components was obtained. We observe a clear dependence of this process on the type of HP. In the case of the FS LIP, the blue shift was suppressed, while in the case of NS LIP we obtained the smallest red shift. For low orders of harmonics, the red/blue shifts are bigger than for higher orders, which could not be explained by a simple model, where only spectral components of leading part of the pump pulse play a decisive role in HHG.

In addition to the findings reported in^46^, we showed the spectral broadening and the appearance of two, redder and bluer, spectral components at two pulse durations of the chirped pulses. Also, in the case of the low fluence FS HP we found the absence of the blue-shifted harmonics generation, which contradicts previous observations^16,46,53,54^. Thus our studies of the NLO properties of two copper-contained perovskites allowed analyzing HHG using the femtosecond, picosecond, and nanosecond heating pulses and demonstrating the specific spectral properties of generated harmonics in the case of two groups of chirped pulses.

## Discussion

The perovskite samples were prepared in the form of the films on the glass substrate with a thickness of a few tens micrometers. The glass substrate at given experimental conditions does not produce the harmonics by any means. Such kind of targets usually requires a lot of efforts to get a stable HHG and to collect enough statistics due to the fast removal of the material by the heating pulses after just a few laser shots. But this is not the case of the studied perovskite samples when, surprisingly, we easily obtained a stable and strong HHG yield for all types of heating pulses (ns, ps and fs), which is unusual feature. So this is a practical reason in the use of the perovskite materials for HHG. Additionally, in general, there are no ways to apply the bulk materials like pure or complex molecular compounds for harmonics generation (apart from the newly developed approach of HHG in solids, which has its pros and cons since this method has a lot of limitations). We believe that HHG from laser-induced plumes is the realistic method to avoid many limitations and to extend the range of materials studies to any species being presented in a plasma form.

The knowledge of the optical nonlinearities related with HHG can be further used in the case of the application of thin perovskite films as the medium for harmonics generation during propagation of the short laser pulses through such films. Another interesting field is the analysis of the resonance enhanced processes allowing the growth of single harmonic yield in the case of the presence of some components, which previously have demonstrated this effect being presented in a pure form in plasma. The modification of spectroscopic properties of the molecules compared with the atoms comprising those molecules could be another interesting application of HHG in the LIPs produced on the surfaces of different perovskites.

Overall, HHG in plasma serves as a method for the analysis of the spectroscopic properties of materials. The high-order nonlinear spectroscopy of ablated species allows defining the yet reported data about the ionic transitions possessing strong oscillator strengths. Notice that the formation of conditions for the most effective conversion of IR pulses towards the XUV range is just a practical aspect of the application of this technique. In the case of the HHG in gases, argon is used as a medium for frequency conversion in most cases. The tasks of the studies of this gas, as well as other few noble gases, are out of the agenda of the researchers. Meanwhile, HHG in plasmas provides the opportunity in the analysis of different materials through laser-plasma interaction. This advantage in use of almost all non-radioactive solids of periodic table in their plasma state for HHG allowed the demonstration of the methods like resonance enhancement of single harmonics, quasi-phase-matching of the groups of harmonics in different ranges of XUV, diversity in the nonlinear optical response of the small-sized species of the variable dimension characteristics (clusters, quantum dots, large nanoparticles), etc. Those features hardly one can expect in the case of the gases. Perovskites are just one of the objects of such studies, which allowed the demonstration of the specific properties of harmonics like broadening, blue- and red-shifts, relatively high conversion efficiency, etc.

One has to maintain the conditions when studied perovskite molecules are not destroyed during ablation and in the plasmas that are produced by the interaction of heating pulses of different duration. Below, we describe the supporting experimental data confirming their survival in the plasma area during propagation of the driving pulses. We also compare our findings with previously published results and analyze some characteristics of the plasmas, which are changing while the target is heated with pulses of different time duration.

Laser-induced plume in general may contain the fragments of original material alongside the intact nanocrystallites, as well as the ionized components and elemental atoms of the original compound. Meanwhile, at “optimal” ablation there are much better chances for perovskites to survive. Thus in our case at “optimal ablation” when no plasma emission was detected we had a plasma comprising dominantly the intact perovskite molecules. Our SEM studies of the surfaces of ablating perovskites were performed to analyze the structural properties of films. These images showed the irregularities of surface structure, which is characteristic of polycrystalline perovskites. Notice that the debris can demonstrate the crystallographic features due to some aggregation of the hot material on the substrate. So this method cannot be considered as a proof of the survival of intact ablated species. The level of disintegration is hard to determine. The option could be the mass-spectrometry of plasma. However, our facility did not allow the simultaneous analysis of the HHG and mass-spectrometry of LIP.

Our studies show the general conditions when the perovskites become presented in plasma without significant morphological modifications. The generality of this approach and similarity of the conclusions for simple and complex targets allow us assume the large probability of the survival of studied samples during laser ablation.

In our experiments, the plasma conditions in the case of using the heating pulse of different duration were modified to achieve the best characteristics of HHG yield (maximal cut-off, best contrast of harmonics signal to plasma emission, maximal yield of harmonics). Laser-induced plasma naturally consists of the mixture of the neutral and ionized fragments of target’s material. Their relative content depends on the heating pulse fluence and pulse duration. In the previous study^55^, the transmission electron microscopy analysis of deposited debris during laser ablation has confirmed a survival of the complex carbon structures (carbon nanofibers, nanotubes, diamond nanoparticles, and C_60_) in the case of the optimal conditions of ablation, which allowed generation of strong harmonics. Other reported studies^56,57^ confirmed the presence and distinguishable contribution of complex molecular components during HHG in the LIPs produced by the nanosecond heating pulses.

The proper simulation of this process is hindered due to the necessity in taking into account a lot of factors. Notice that the perovskites show some features similar to the carbon-containing nanostructures (fullerenes, carbon nanotubes and nanofibers, diamond nanoparticles, etc.) from the point of view of the harmonics spectral broadening^57^. These empirical observations still yet find the explanation.

Our studies were related to the mechanism of the appearance of red and blue components. However, together with these shifts of the central part of harmonics one can admit the presence of the wavelength related to the integer of the driving wave (i.e. exact positions of harmonics on the wavelength range corresponding to the ω/N frequency; N is the order of harmonic). Our studies show both the shifted parts of harmonics alongside the un-shifted part. The broadening of these harmonics can be attributed to the presence and overlap of two components. This is a commonly observed and reported observation of the harmonics in the case of HHG in plasma.

Our present studies of the influence of the fluence of heating pulse on the decay of harmonic yield showed how this process notably modifies at higher fluencies resulting in almost entire disappearance of harmonic emission. The growth of heating fluence caused the disintegration of perovskites, appearance of strong plasma emission, growth of the concentration of free electrons in the plasma plume, and correspondingly, decay of harmonic yield caused by the phase-mismatch induced by the excess of the free electrons. Meanwhile, at small fluencies of heating pulses, the pure harmonic spectra were observed without any manifestation of the plasma emission (Figs. 2, 3, and 4), which is a direct confirmation of the presence of intact perovskites in LIPs at small fluencies followed by their disintegration at larger fluencies. Thus the heating pulse fluence is gradually increasing from the “optimal” to the one at which the overheated plasma causes the degradation of the HHG process. Summing up, the optimal HHG conditions correspond to the “mild” ablation regime leading to the formation of the weakly ionized plasma, which mostly contains the ejected material of the target.

In addition, the plasma characteristics are changing at the conditions when the target is heated with the pulses of different duration, i.e. by using the NS, PS, and FS pulses. In brief, the comparison of HHG yields in the case of those heating pulses showed that pulse duration influences the cut-off of harmonics. The plasma expansion and its density vary for different durations of heating pulses. The variation in the density of the plasmas consisting of the atoms, ions, and NPs affects the yield, cut-off order, as well as the shape of the plateau-like distribution of harmonics. In our study, we demonstrated that the plasma plumes produced on the surface of perovskites by nanoseconds pulses enhances the harmonic cut-off.

In the case of metals, the laser ablation and formation of plasma using NS and FS pulses were reported earlier in^51^. The population of LIP and dynamics of plasma spreading in those two cases also depend on such laser parameters as the wavelength and pulse energy, as well as the ambient environment conditions. Particularly, the NS-induced LIPs possess the spherical shape of expansion, whereas, the FS-induced LIPs show the cylindrical expansion. Verhoff et al.^51^ showed that FS LIP provided narrower angular distribution of ions and evaporated mass in comparison with NS LIP. Similarly, for picosecond pulses induced plasma, the expansion angle is smaller than in the case of the NS LIP.

One of the practical advantages of using NS pulses induced LIP is the absence of the limit in the formation of the suitable time delay between the heating and driving pulses. This advantage is especially important in the case of large/heavy species like fullerenes, perovskites, small clusters and quantum dots, and to some extent, nanoparticles, since the formation of the delay by optical methods (e.g. extended optical lines) in practice is limited to ~ 100 ns, which is physically corresponds to the additional path of the driving pulses for more than 30 m.

Notice that, for some targets like metals, the shorter pulses allowed improving the harmonic yield and cut-off (for example^58^). However, in the present studies, the maximum cut-off up to 19H was achieved in the case of NS heating pulses. This might be due to the higher fluence (9 J/cm^2^) of NS pulses than in the case of fs pulses (0.6 J/cm^2^). Therefore, the additional advantage of NS pulses is that they create the stable plasma plumes for a longer timescale than what can be achieved using the FS heating pulses. Additionally, in the case of FS pulses, large intensity can disintegrate the complex molecules like perovskites, while the application of NS pulses at similar fluence and significantly smaller intensity allows maintaining the intact molecules during evaporation from the targets. In our case, the application of NS LIP led to the enhancement of the higher-order harmonics in the range of the 9H to 17H, compared to the FS LIP. Thus, the optimization of the LIP for HHG in the case of different targets may show the variable behavior for NS, PS, and FS ablating pulses.

The question may arise on how the results from perovskite materials are different from non-perovskite materials? The materials other than perovskites such as bulk metals, nanoparticles of different materials, carbon-containing nanostructures, etc. show the extension of cut-offs based on their ionization potential and pondermotive potential. Earlier, various materials were explored as the suitable plasma plumes to analyze their ability to generate the higher-order harmonics using different driving laser pulses and heating pulse parameters. Particularly, the Mn plasma allowed highest harmonic cut-off (> 100H) among all other plasma plumes.

To the best of our knowledge, previous studies of HHG in perovskites were limited by a few publications^45,46^ in the case of the Ni-doped CsPbBr_3_ perovskite nanocrystals. This is a new field of studies, which allows further development of the laser-induced high-order harmonic spectroscopy of materials. In present study, we show that other studied perovskites ((CH_3_NH_3_)_2_CuCl_4_ and (CH_3_NH_3_)_2_CuBr_4_) allow generation of strong harmonics in the lower-order range, i.e. for the range of 9H to 21H. These harmonics can be applicable for the nonlinear spectroscopy and autosecond studies in the selected regions of XUV. The present study also demonstrates a pathway to explore the novel perovskite thin films for the generation of higher-order harmonics using different driving pulse durations having negative and positive chirps. We demonstrated the tunability of harmonics, which could be achieved by changing the chirp of pulses.

The artificial change of the of the chirp of pump radiation has successfully been applied in previous experiments to tune the harmonics generated in the laser plasmas prepared on the surfaces of various solid-state materials^59–61^. This technique allowed achieving a resonance-induced single-harmonic enhancement in the cases of some laser ablation plumes^62^. In the case of plasma produced on the surface of various solid-state materials, there is a higher probability to find a proper target for which the fulfillment of multiphoton resonance conditions in the XUV range can lead to an enhancement of the harmonic yield. At the same time, the near absence of the resonance enhancement of harmonics in laser-gas jet experiments can be explained by a decreased probability of such coincidental resonance between the atomic or ionic transitions and the harmonic wavelengths in the case of a few gases commonly used during the HHG experiments.

In our studies, we showed that the proper chirp variations can lead to an improvement of the phase matching conditions, narrowing or broadening the harmonic bandwidth, and, in some cases, harmonic cut-off extension. The studied perovskite plasmas did not allow the observation of resonance-induced enhancement due to the absence of the ionic transitions possessing high oscillator strength. However, this process could be observed in other perovskites provided they possess strong ionic transitions.

The conclusion about the improvement in the phase-matching of harmonics and driving waves was made on the basis of the experimental observations. The narrowing and broadening of harmonic bandwidth are shown in Figs. 2, 3, and 4. We also observed the enhancement of the harmonic yield in some cases when the chirped pulses were used. This effect is available only in the case of the improvement of the phase-matching conditions.

Chirp control of the pump laser allows one to achieve an optimal relation between the quasi-resonance conditions, reabsorption, and induced self-defocusing leading to an enhancement of the harmonic yield, similar to what was demonstrated in our present studies. The quasi-resonance between some specific harmonic and fundamental waves can be realized in the vicinity of some ionic transitions possessing strong oscillator strength. The tuning of harmonic wavelength can create the conditions when the closeness of harmonics to those transitions can change the refractive index, depending from which side those harmonics approaching to the ionic resonances. This effect was analyzed in the case of the metal plasmas where the single-harmonic enhancement was frequently reported. In the case of molecular structures, this effect is diminished^63,64^, probably due to a decrease of the oscillator strengths of the transitions under consideration. At the same time, the re-absorption can play important role at these conditions. The interplay between those two processes can lead to either increase or decrease of the harmonic yield depending on various conditions.

To decisively answer to the question on how and up to which extent the quasi-resonance attributed to the shift of the wavelength of generating harmonic influences the yield of this wave one has to consider both macro- and micro-processes. The re-absorption may play a role in the vicinity of the resonances. It is a very complex problem, which has been earlier considered from the point of view of the prevalence of the micro-process^65^. It is still not clear which of two processes plays a decisive role. Meanwhile, the simulation results supporting our claim were presented in^65^.

As already mentioned, previously, the resonance-induced enhancement of a single harmonic was achieved using the chirping of the broadband laser pulses. This led to the tuning of harmonics toward the appropriate transitions possessing strong oscillator strength. The tuning of harmonics became available due to the redistribution of the laser spectrum along the main pulse and preferential harmonic generation at the leading part of the pulse. The defocusing properties of plasma weakened the trailing part of the pulse. That is why the leading part of the chirped pulse was mainly responsible for the harmonic tuning. We did not change the spectrum of the fundamental radiation, but instead changed the spectral distribution inside the pulse by controlling the chirp of the laser radiation.

Splitting and tuning of harmonics could be explained by deviation from the assumption of the main role of the leading front of laser pulse as a source responsible for the variation of the spectral position of harmonics when the ionization of studied samples by the driving pulses can cause a significant change in the whole dynamics of laser-plasma interaction. Some simulations of this process were carried out in the case of HHG in gases. The spectrum of harmonics generated and propagated in ionized noble gas has been analyzed using one-dimensional wave propagation equation^66^ They analyzed a relation and influence of laser parameters and gas pressure on the splitting of harmonics. The result shows that the spectral lines of harmonic become broadened and then split into two peaks when the laser intensity is strong enough to ionize the noble gas. They also investigate the influence of ionization on the propagation and spectral effects of a few-cycle ultrashort laser pulse. It is found that when the fractional ionization is weak, the production of higher spectral components makes no difference. However, when the two states are essentially depleted before the peak of the laser pulse, the impact of ionization on the higher spectral components is very significant.

In our case, the ionization of the studied molecular structures can also lead to similar effects predicted by abovementioned study. However, in our case some additional factors like pulse duration and threshold of ionization can play a decisive role, which can change the whole pattern of harmonic tuning and splitting.

Our studies showed in some cases the variations of the harmonics obtained from two different perovskite LIPs under equal experimental conditions while using the variable chirps of laser pulses. LIP is a complex medium, especially from the point of view of its NLO properties. The behavior of laser ablation changes considerably, depending on the elemental composition, equation of state, ionization potential, and cohesive energy of the material. The processes that determine harmonic generation from the plasma plume may involve various factors that are not considered for gas harmonics. The harmonic spectrum can be both broadened and narrowed by frequency chirp of harmonics depending on the target material, intensity, and focusing conditions of the driving pulse. Particularly, a harmonic cut-off extension by optimization of the chirp parameters of femtosecond pulses was observed in the case of the HHG from the perovskite LIPs.

The difference between two perovskite materials was shown through the HHG under the influence of the strong electromagnetic field. The observed differences in HHG spectra between two materials do not allow clearly identify the role of the Br and Cl atoms. The main effect was pronounced by variation of the chirp/pulse duration and HP type, which allowed observing the difference in HHG spectra between two materials.

Overall these studies demonstrate the importance of chirp parameters even in the case of the relatively long femtosecond pulses to achieve an extended harmonic cut-off and higher conversion efficiency compared with the chirp-free pulses. The range of tuning was restricted by the bandwidth of the laser pulse. In present studies, we analyzed the tuning of harmonics in a narrow range, while keeping optimal perovskite plasma conditions for the HHG.

## Conclusions

We reported the study of laser chirp effect and heating pulse effect in the case of high-order harmonics generation during two-color pump of the laser-induced plasmas produced on the surfaces of two perovskite targets (MA_2_CuCl_4_ and MA_2_CuBr_4_). We found a difference in the influence of the sign of chirp and the pulse duration on the spectral and energy properties of the high-order harmonic spectra. Regardless of the choice of the duration and fluence of heating pulses, the positively chirped 70 fs driving pulses caused the spectral broadening and the appearance of two, redder and bluer, spectral components. The relative position of these components compared with the chirp-free 35 fs induced harmonics depended on the type of heating pulses and the chosen material. It was found that, in the case of negatively chirped pulses, the blue shift of harmonics does not appear once the plasma became formed by the femtosecond pulses. This peculiarity can be attributed to the smallest used heating pulse fluence, since in the case of nanosecond and picosecond ablation the blue shifts were presented. The variation of the laser chirp allowed considerable tuning of the harmonics towards the shorter wavelengths (particularly, by ~ 2.5 nm for the 13H and 15H). The variations of harmonic spectra demonstrated in these studies can be used in the high-order nonlinear spectroscopy of different materials.

## Method

The upper panel of Fig. 1 shows the schematic for LIP analysis via HHG experiments. The target and XUV spectrometer chambers were maintained at vacuum (~ 3 × 10^–5^ mbar). The heating pulse was focused by a 300 mm focal length spherical lens on the surface of targets. The femtosecond probing pulse (800 nm, Spitfire-Ace, Spectra-Physics) was focused by a 400 mm focal length spherical lens inside the LIP produced on the surfaces of ablated targets. The high-order harmonics were analyzed using the XUV spectrometer. The HHG spectra were collected using a CCD camera. Apart from the single-color pump (800 nm), we used the two-color pump (800 nm + 400 nm) of LIP. The 0.2 mm thick BBO crystal was placed inside the vacuum chamber on the path of the focused femtosecond pulses (35 fs, 800 nm, 0.6 mJ) to generate a second harmonic (400 nm), which allowed us to generate the harmonics using TCP scheme. The conversion efficiency to the second harmonic was measured to be ~ 5%. The polarizations of the fundamental and SH beams were perpendicular to each other. These two pulses were focused inside the LIP at a distance of ~ 0.2 mm above the target surface. The intensities of the fundamental chirp-free pulses (35 fs) and the SH at the focal spot were ~ 3 × 10^14^ W cm^-2^ and ~ 1.2 × 10^13^ W cm^-2^, correspondingly.

The chirps, signs, and PP duration were controlled by changing the distance between gratings inside the optical compressor. The pulse duration was varied from 35 to 70 fs and 130 fs. The bottom panel of Fig. 1 shows two groups of spectra of the used 35, 70, and 130 fs pulses at different signs of chirp. Chirped and chirp-free pulses possessed the same spectral components (Fig. 1, bottom panel). However, their distribution along the laser pulses significantly differed from each other. The blue and red components were moved towards the leading and trailing parts of the pulses, respectively, in the case of the negatively chirped pulses. In the case of positively chirped pulses, the red component was moved towards the leading front of the pulse, while the blue component of the pulse spectrum was concentrated at the trailing part of the pulse.

The perovskite materials (MA_2_CuCl_4_, MA_2_CuBr_4_) were prepared in the form of a coating on the glass substrate with a uniform thickness (1 mm) and placed inside the vacuum chamber for further ablation. MA_2_CuCl_4_ and MA_2_CuBr_4_ perovskites were synthesized from ethanol solutions with the desired stoichiometry. For MA_2_CuCl_4_, the precursors were mixed in ethanol, stirred for 2 h at 60 °C, and left to crystallize overnight in an ice bath. The product was recovered by filtration, and dried at 60 °C for 12 h in a vacuum oven. The MA_2_CuBr_4_ was obtained after complete evaporation of the solvent. The details of the synthesis of these perovskites are described elsewhere^67^. To fabricate thin films, 1 M solution of above perovskites was drop-coated on the glass substrate and then annealed at 70 °C for 1 h.

The femtosecond, picosecond, and nanosecond pulses were used as HP for the ablation of the target. The fluencies of those pulses were ~ 0.6 J cm^-2^ (in the case of FS HP), ~ 2 J cm^-2^ (in the case of PS HP), and ~ 9 J cm^-2^ (in the case of NS HP). In the case of using FS and PS HP, an optical delay line was used, which allowed the maintenance of the fixed delay (70 ns) between the HP and PP. The PS HPs were taken by the partial division of the uncompressed 200 ps PP beam before entering the compressing stage, while the FS HPs were split at the output of the optical compressor.

In the case of the NS HP, an approach similar to the one described in^55,56^ was used. The delay between NS HP from Nd: YAG laser (5 ns, 1064 nm, 10 Hz; Q-Smart, Coherent, USA) and FS PP was varied using the digital delay line and controlled by a delay generator (DG535, Stanford Research Systems). This approach allowed us to remove the limitation for the delay formation in a broad timescale in contrast with the optical delay line. In the presented experiments, the optimal delay for NS pulse was found to be 400 ns, while in case FS and PS HP, the 70 ns delay was applied.

## Data Availability

The datasets used and/or analyzed during the current study available from the corresponding author on reasonable request.
